# Hatchability and survival of oncomiracidia of *Paradiplozoon ichthyoxanthon* (Monogenea: Diplozoidae) exposed to aqueous aluminium

**DOI:** 10.1186/s13071-016-1706-z

**Published:** 2016-07-28

**Authors:** Beric M. Gilbert, Annemariè Avenant-Oldewage

**Affiliations:** Department of Zoology, University of Johannesburg, Johannesburg, South Africa

**Keywords:** Heavy metals, Larval development, Metal accumulation, Toxicity, Pollution, Parasite indicators

## Abstract

**Background:**

Monogenea is a diverse group of ectoparasites showing great potential as sentinel organisms for monitoring environmental health. Exposure to metals negatively affects infrapopulations of monogeneans and exposure to aluminium has been found to negatively impact the survival of gyrodactylids.

**Methods:**

Samples of infected host fish, the smallmouth yellowfish *Labeobarbus aeneus* (Cyprinidae), were collected from the Vaal Dam, South Africa and transported back to the laboratory in dark 160 l containers. Eggs of the monogenean *Paradiplozoon ichthyoxanthon* infecting *L. aeneus* were collected and exposed to varying concentrations of aluminium along with a control group in static tanks. The eggs were checked every 24 h and hatching commenced 13–14 days after exposure. Water samples were taken from exposure tanks and acidified for analysis of Al levels with inductively-coupled plasma mass spectrometry.

**Results:**

Hatching of eggs was variable between exposures, and in 30 μg Al/l and 60 μg Al/l was found to occur before eggs in control beakers, whereas, exposure to 120 μg Al/l delayed hatching and reduced hatchability. Survival of hatched oncomiracidia was concentration dependent and negatively correlated with aluminium concentrations. Lowest survival was recorded for 60 μg Al/l and 120 μg Al/l where all larvae died shortly after or during hatching. Normal development of embryos of *P. ichthyoxanthon* within eggs exposed to all doses of aluminium indicates that the egg shell is moderately impermeable to metals and inhibits movement of aluminium across the shell and interacting with developing embryos.

**Conclusions:**

Higher larval mortality rate in 120 μg/l exposure can be related to aluminium crossing the egg shell in the late stages and causing death of unhatched yet fully developed embryos, possibly due to changes in the permeability of the egg shell as embryos neared developmental completion. Accelerated death of oncomiracidia after hatching indicates sensitivity toward high concentrations of aluminium.

## Background

The sensitivity of parasites in general toward metals is poorly documented, complicating the interpretation of field studies and obscuring the identification of sensitive taxa which can serve as bioindicators for monitoring environmental degradation [1, 2]. Studies have suggested that some parasites are useful biological indicators of environmental health [3]. Most of these studies have focused on the use of parasites as accumulation indicators, especially endoparasites [4–6]. Monogenean parasites have been shown to be sensitive toward metal pollution [7] and changes in infection biology of this group have been identified for a number of different species [8–10]. In terms of pollution indication, all investigations thus far have focused on infection level alterations with few studies documenting accumulation patterns in this group. Exposure to pollution by metals [11, 12], acidification [7, 13] and pulp and paper mill effluent [14, 15] have been found to negatively affect prevalence, mean intensity and abundance of monogeneans, while the opposite has been found to occur in the presence of hydrocarbons and eutrophication [16, 17]. In terms of metal toxicity toward monogeneans, a number of studies have documented the potential use of aluminium as a parasiticide for the treatment of adult gyrodactylid infections in fishes under laboratory conditions [18–20]. These studies have noted that exposure to elevated aluminium concentrations is able to completely eliminate infections with little toxicity toward the hosts. However, no investigations have documented the toxicity of aluminium or other heavy metals toward diplozoid monogeneans under laboratory conditions.

The present work forms part of an investigation on the responses of the diplozoid, *Paradiplozoon ichthyoxanthon*, toward environmental pollution and, therefore, its use as a sentinel organism in the Vaal River system. An earlier field investigation [10] had documented the local extinction of this parasite at a site in the Vaal River, below the Vaal River Barrage, where elevated levels of metals and poor water quality have been identified for a number of years [21, 22]. Dissolved aluminium concentrations at the Vaal River site, where the parasite is absent, were 23 times higher compared to levels at the Vaal Dam where parasites are present [10]. Further to this, a study was done to describe the microhabitat biology of the parasite in the Vaal Dam in relation to the physical water quality variables at the site, where changes in microhabitat selectivity were the result of seasonal changes and were not driven by water quality [23]. This study is therefore a continuation of this work and aimed to describe the effects of exposure of eggs and larval stages of *P. ichthyoxanthon* to aluminium. Larval stages of the parasite were selected as this developmental stage is responsible for transmission of parasites to new hosts. Therefore, we examined the effects that aluminium exposure would have on the hatchability of the eggs and survival of oncomiracidia of *P. ichthyoxanthon*.

## Methods

### *P. ichthyoxanthon* egg collection

*Labeobarbus aeneus* infected with *P. ichthyoxanthon* were collected from the Vaal Dam using gill nets and maintained on UJ Island (26°52′33.62″S, 28°10′25.76″E) in 160 l holding tanks containing aerated borehole water. From here the fish were anaesthetised with 100 ml 2-Phenoxy Ethanol (Sigma-Aldrich, Steinheim, Germany) in 160 l water and transported back to the Parasitology Laboratory at the University of Johannesburg. In the laboratory fish were kept in the dark and eggs were collected daily when water changes were done with reverse osmosis (RO) water. According to MacDonald & Jones [24] laying of eggs by diplozoids increases during periods of darkness. Parasite eggs were collected by filtering water through a Visser Sieve apparatus [25] (25 μm mesh). Filtrate was collected from the funnels in 500 ml beakers and then aliquots of filtrate were examine in a glass Petri dish with a Zeiss DV4 stereomicroscope. Eggs of *P. ichthyoxanthon* were identified, isolated and collected with a triple zero Camel’s hair paintbrush and placed into a clean Petri dish containing aerated RO water. Collected eggs were kept in fresh, aerated RO water for 24 h before exposure experiments were commenced.

### Exposure bioassays

Static bioassays were performed using varying nominal concentrations of aluminium (0–120 μg/l). A stock solution of 10 mg Al/l was made up by weighing out 1.07 g aluminium nitrate (Sigma-Aldrich, St Louis, United States of America) and dissolving in 500 ml RO water. The 10 mg/l Al stock was aerated for 24 h before exposure experimentation commenced to negate any possible effects that Al may have on the dissolved oxygen levels of the RO water. Exposure media were made up by serially diluting 10 mg/l stock solution with fresh aerated RO water to a final volume of 150 ml per beaker. Exposure assays were prepared in triplicate in acid washed glass beakers and three eggs were placed into each of the replicates. Exposure levels of aluminium were selected according to the environmental relevance to the Vaal Dam and Vaal River site assessed in Gilbert and Avenant-Oldewage [10], and slightly higher for comparison to previous studies conducted on exposure of a number of species of *Gyrodactylus* to varying aluminium levels [18–20]. Reference to metal concentrations in the study is done on the basis of nominal concentrations at the beginning of the exposure. Experiments were performed in environments with constant photoperiod and room temperature at 14 h light and 10 h dark, and 23 °C, respectively. Exposure tank temperatures were therefore maintained at a temperature of 20.8 °C (± 0.21). Eggs and physical water quality variables (*vis*. pH, temperature, conductivity, dissolved oxygen and oxygen saturation) were measured every 24 h to observe if any noticeable changes in development of the embryos and water quality occurred. A total of 55 eggs of *P. ichthyoxanthon* were exposed to varying concentrations of aluminium for a period of 19 days, namely: RO water (control); 7.5 μg Al/l; 15 μg Al/l; 30 μg Al/l; 60 μg Al/l and 120 μg Al/l. Exposure of the eggs was continued until hatching had stopped to determine if the levels of metal affected the hatchability of the eggs as well as the survival of oncomiracidia. Exposures were concluded at day 20 when hatching of eggs ceased and all oncomiracidia had perished. Larval development was monitored throughout the duration of the exposure. Development of oncomiracidia of *P. ichthyoxanthon* was monitored following Avenant-Oldewage & Milne [26]. Eggs collected from tanks were identified at the oncocyte developmental stage, indicating an approximate age of about 3 days post laying [26]. In their study development of *P. ichthyoxanthon* began with the oncocyte (day 3), followed by the appearance of the oncoblast (day 5) as a darkened area in the centre of the egg. This was then followed by the appearance of the eye-spots (day 16) along with longitudinal body contractions. The operculum, ciliary movements, pharyngeal movement and movement of the clamps were the last features to become noticeable (day 19). Movement of fully developed embryos were used to monitor vigour during late developmental stages, when embryos were fully formed. Movements of the embryos were assessed using a stereomicroscope by viewing for approximately one minute and observing the frequency of clamp, body and ciliary movements. Once hatched, the ciliary movement and response to agitation with a Pasteur pipette were used as indicators of the vitality and therefore survival of oncomiracidia. If no ciliary movements were seen and the larvae did not respond to agitation by a pipette, it was concluded that at this point, larvae were dead.

Subsamples of exposure media were taken every 24 h and acidified with 231 μl concentrated nitric acid (65 % Suprapur; Merck, Darmstadt, Germany) from each of the tanks for determination of the actual aluminium levels in each tank. The levels of the metal were determined with an inductively-coupled plasma mass spectrometer (NexION® 300 Series; Perkin Elmer, Waltham, USA). Rhodium (100 μg/l) was used as an internal standard and was added online during analysis using an internal standard mixing kit.

### Statistical analysis

Analysis of the actual concentrations of aluminium, hatchability of eggs and survival of oncomiracidia per aluminium concentration was assessed using SPSS V.22 for Windows (Statistical Package for the Social Sciences, SPSS Inc., USA). Homogeneity of the data for aluminium analysis was tested using a Shapiro-Wilk test. Trends in temporal variances of metal levels in exposure assays during the course of the experiment were assessed using a Kruskal-Wallis test. Comparison of the differences between concentrations over the period of the study were assessed using a one-way analysis of variance (ANOVA) and Tukey tests. Spearman’s correlation was used to determine the relationship between time and the levels of aluminium eggs and larvae were exposed to. Comparison of mean number of eggs hatching, the percentage of living and dead oncomiracidia, and percentage of actively swimming and moribund larvae of the total number of hatched individuals between different exposure media was assessed using ANOVA and Tukey tests. Comparison between the percentage of live and dead oncomiracidia between exposure media and days was done using Pearson’s chi-square and Kruskal-Wallis H-test respectively. The relationship between the number of eggs hatching and number of living oncomiracidia between different exposure concentrations was assessed using a Pearson’s correlation test. Survival of oncomiracidia after hatching was assessed statistically using the Kaplan-Meier method. A log-rank analysis was done to test the significance in differences of oncomiracidium survival between different aluminium concentrations. All significance limits were set at *P* = 0.05 to determine if differences in variables compared were significant or not.

## Results

### Aluminium analysis

**Fig. 1:**
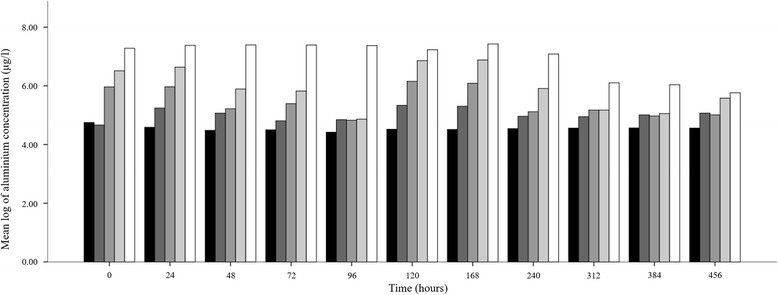
Bar charts indicating the mean log of aluminium levels in exposure tanks over the period of the study. Concentrations are indicated by varying scales of grey (*black*: 7.5 μg/l; *dark grey*: 15 μg/l; *medium grey*: 30 μg/l; *light grey* 60 μg/l; *white*: 120 μg/l)

Analysis of Al levels in exposure assays indicated that metal concentrations decreased slightly over time for the first 96 h and following addition of fresh Al solution to all exposure media, concentrations decreased but did not deviate significantly over the remaining period of the study (Fig. 1). Due to the insignificant change in aluminium levels following 96 h period subsequent samples were taken at 72 h intervals for the remainder of the study. Normality of the data was assessed and found to be non-normally distribution (Shapiro-Wilk *t* = 0.700, *df* = 15, *P* < 0.0001), therefore, data were normalised through logarithmic transformation. Comparison of the transformed data indicated significant differences between the different aluminium concentrations (ANOVA *F*_(4, 160)_ = 90.26, *P* < 0.0001), whereas, concentration variances between 24 h intervals were not significantly different (Kruskal-Wallis H-test *χ*^2^ = 15.07, *df* = 10, *P* = 0.129). Correlation analysis of the data further indicated that this was weakly negative and insignificant between the time samples were taken and the concentration of the metal (*r* = -0.146, *P* = 0.008).

**Fig. 2:**
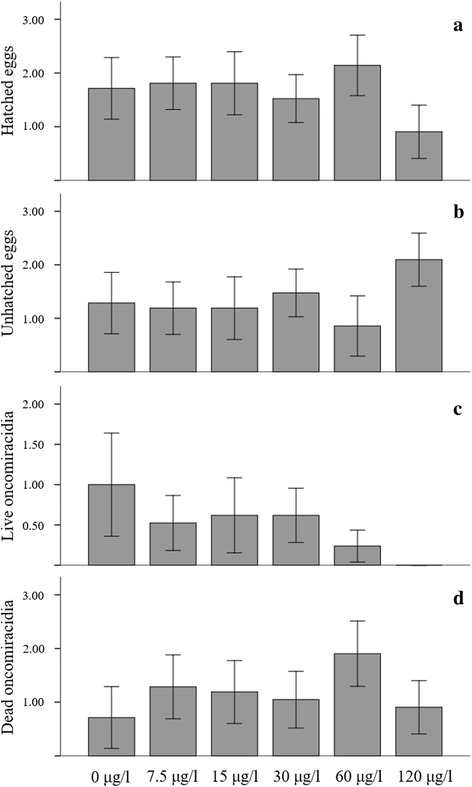
Bar charts indicating the mean number of hatched eggs (**a**), unhatched eggs (**b**), live oncomiracidia (**c**), and dead oncomiracidia (**d**) of *Paradiplozoon ichthyoxanthon* per exposure concentration. Error bars indicate 95 % confidence intervals

Hatchability of eggs and longevity of oncomiracidia of *P. ichthyoxanthon* exposed to a range of aluminium concentrations varied (Fig. 2). Effects of the metal on hatchability (Fig. 2a and b) and oncomiracidium survival (Fig. 2c and d) were concentration dependent. Of the total 55 eggs exposed for the full duration of the study (19 days) 86 % of eggs hatched (*n =* 46) while 16 % (*n* = 9) did not and differ significantly (Pearson Chi-square test *χ*^2^ = 28.15, *df* = 15, *P* = 0.021). The highest number and percentage (Fig. 2a) of eggs to have hatched were those from the 60 μg Al/l exposure, this was followed (in descending order) by the 7.5 μg/l = 15 μg/l > 0 μg/l > 30 μg/l > 120 μg/l, where the lowest number of eggs hatched. Hatching time was also staggered between the different aluminium exposures and first occurred in the 30 μg/l and 60 μg/l exposure on day 13 of the experiment. This was followed by eggs in control, 7.5 μg/l and 15 μg/l exposures (day 14), whereas, eggs exposed to 120 μg/l (day 16) where the last to start hatching. Compared to the hatching of eggs in controls, hatching time was thus accelerated in the 30 μg/l and 60 μg/l exposure and delayed in the 120 μg/l exposure. The number of eggs hatching was significantly different (ANOVA *F*_(5, 113)_ = 2.88, *P* = 0.017) between different exposure media. However, closer examination indicated that only differences between 60 μg/l and 120 μg/l exposures were significant (Tukey test *F* = 1.24, *P* = 0.007), whereas, comparisons of the hatchability between other exposure media were not significantly different (all *P* > 0.05). Furthermore, there was no significant difference in the number of eggs hatching between replicates (ANOVA *F*_(2, 116)_ = 0.15, *P* = 0.861), while, the number of eggs hatching between different days was found to be significantly different (ANOVA *F*_(6, 112)_ = 32.18, *P* < 0.0001).

Larval development was not affected by exposure to aluminium. All embryos became fully developed with clearly visible eye-spots and clamps. Within the first 96 h, the yolk contents of the eggs became dense and obscured visualisation of the oncocyte, but slight movement of developing larvae could be seen as wave-like motions within the yolk mass, particularly at the anterior end of the egg, below the level of the operculum. This was similar for all eggs in different exposure media. This was followed by visualisation of the oncoblast within the centre of the egg. This was seen as a less dense and somewhat lighter coloured region of the contents of the eggs and wave-like movements of early embryos became pronounced from the eighth day till the eleventh day of incubation. By the twelfth day eye-spots could be seen clearly, obvious movements of larvae were noticed and cilia were seen to beat constantly in all exposures. Finally by the thirteenth day the clamps and fully developed oncomiracidia were observable. Over this period the operculum become progressively more visible and distinct.

At this point hatching commenced and varied between different exposures, first occurring in the 30 μg/l and 60 μg/l exposures. The frequency of larval movements within eggs increased as hatching commenced, where vigorous movements of the whole body of the larvae increased and were most obvious just prior to hatching. The operculum of the eggs became increasingly visible. Over this period, ciliary movements were constant. The development of larvae was similar in the various exposure media. Movement of larvae within eggs were found to comprise of rapid rotation of oncomiracidia within the eggs, occurring frequently along with numerous contractions and extensions of their bodies, in a way that appeared as if larvae were forcing their bodies against the operculum. Noticeable differences in the movement of unhatched oncomiracidia were seen in the 120 μg Al/l exposure, where larvae in this exposure moved less frequently than those in other exposures. Furthermore, mortality within the eggs became apparent as reduced frequency in movement of larvae, until all movements of oncomiracidia within eggs ceased. Frequency of the movement of larvae in the eggs was not measured as it was not specific for exposure to the metal and in the early stages of development was noted to be independent of aluminium toxicity or exposure.

**Fig. 3:**
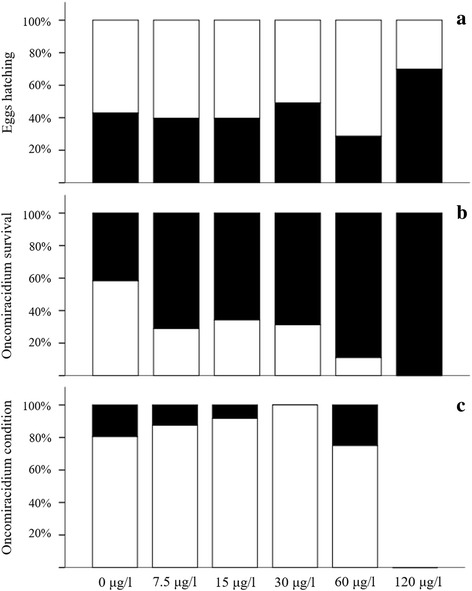
Stacked bar charts showing the percentage of hatched (*white bars*) and unhatched eggs (*black bars*) (**a**), percentage of dead (*black bars*) and living (*white bars*) oncomiracidia (**b**), and percentage of actively swimming (*white bars*) and moribund (*black bars*) oncomiracidia (**c**), of *Paradiplozoon ichthyoxanthon* per exposure concentration

Survival of hatched oncomiracidia was lowest in 120 μg Al/l exposure, with larvae perishing shortly after hatching compared with survival in lower aluminium concentrations (0–30 μg/l). The percentage of living oncomiracidia compared to the percentage of dead ones per concentration is illustrated in Fig. 3b. The highest mortalities were recorded for 120 μg Al/l exposure, where 100 % of all larvae that hatched died. The oncomiracidia from 60 μg Al/l and 120 μg Al/l often perished in close proximity to the eggs shells from which they hatched, indicating that they succumbed shortly after emerging from the eggs. The highest percentage of actively swimming individuals was recorded for the 30 μg Al/l exposure. This was followed by 15 μg Al/l > 7.5 μg Al/l > control > 60 μg Al/l. At 120 μg Al/l 0 % of larvae survived after hatching. The highest percentage of moribund oncomiracidia were those hatching in the 60 μg/l exposure, while the lowest was recorded at 30 μg/l, but this was not significantly different between Al exposures (Kruskal-Wallis H-test *χ*^2^ = 3.84, *df* = 5, *P* = 0.383). The cilia of these larvae were still actively moving but oncomiracidia did not swim even when agitated with a Pasteur pipette. Whereas, those exposed to 7.5 μg/l and 15 μg/l were actively swimming in the beakers and the only difference to the control was in their survival time (Fig. 3c). The percentage of living oncomiracidia was significantly different between the exposures (ANOVA *F*_(5, 36)_ = 3.16, *P* = 0.018). Comparison of the percentage of oncomiracidia which died during the exposures differed significantly with the day of the exposure (ANOVA *F*_(6, 35)_ = 31.15, *P* < 0.0001). Significant differences in the percentage of living (Kruskal-Wallis H-test *χ*^2^ = 13.32, *df* = 5, *P* = 0.021) oncomiracidia were found for 120 μg Al/l exposure only (lowest rank according to Kruskal-Wallis H-test: mean rank 10). This indicates that death of larvae in these exposures as a result of aluminium toxicity was relatively rapid.

Differences in oncomiracidial survival between different aluminium concentrations were significantly different (Kruskal-Wallis H-test *χ*^2^ = 36.08, *df* = 5, *P* < 0.0001). *Post-hoc* analysis of the number of live oncomiracidia indicated significant differences between those in the control and the 60 μg Al/l (*F* = 0.76, *P* = 0.029) and 120 μg Al/l (*F* = 1.00, *P* = 0.001) exposures while comparisons between 7.5 μg Al/l, 15 μg Al/l and 30 μg Al/l to 60 μg Al/l and 120 μg Al/l concentrations and between each other, were not significant (all *P* > 0.05). The highest number of live oncomiracidia was found on day 15 of the exposures. This further differed between the different exposures, specifically 15 μg Al/l and 60 μg Al/l exposures, where the highest number of live oncomiracidia were recorded on day 16. Daily differences in the number of oncomiracidia alive in all of the exposures were significant (ANOVA *F*_(6, 112)_ = 5.04, *P* < 0.0001), with noteworthy differences between days 13 with 15 (*F* = -0.88, *P* = 0.005) and 16 (*F* = -0.76, *P* = 0.026); day 15 with days 18 (*F* = 0.82, *P* = 0.012) and 19 (*F* = 0.94, *P* = 0.002); day 16 and 19 (*F* = 0.82, *P* < 0.0001).

**Fig. 4:**
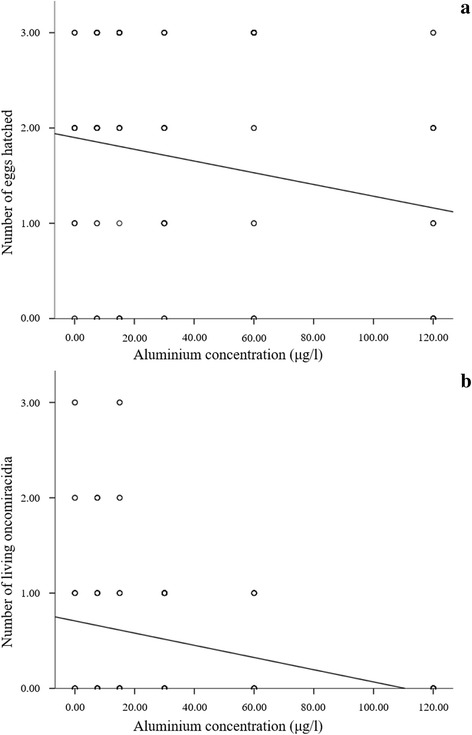
Number of hatched eggs (**a**) and live oncomiracidia (**b**) compared to aluminium concentrations

Results of exposures indicate that the hatchability of eggs and longevity of oncomiracidia of *P. ichthyoxanthon* negatively related to the concentration of aluminium (Fig. 4a and b). Both hatchability (Fig. 4a: y = -6.16^-3^ × *χ* + 1.9, *R*^2^ = 0.048) and survival of oncomiracidia (Fig. 4b: y = -6.41^-3^ × *χ* + 0.71, *R*^2^ = 0.123) decreased as aluminium concentrations increased. This was confirmed by Pearson’s correlation, which indicated significant and strong negative correlations between the number of hatched eggs (*r* = -0.218, *P* = 0.017) and live larvae (*r* = -0.351, *P* < 0.0001) and aluminium concentration.

**Fig. 5:**
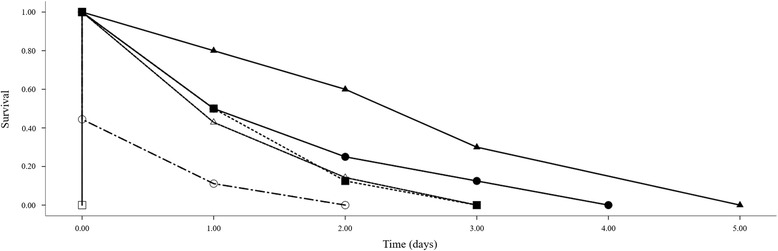
Kaplan-Meier plot of the cumulative survival probabilities of oncomiracidia of *Paradiplozoon ichthyoxanthon* exposed to 0 μg/l (*black triangles*), 7.5 μg/l (*black circles*), 15 μg/l (*black squares*), 30 μg/l (*white triangles*), 60 μg/l (*white circles*) and 120 μg/l (*white square*) of aluminium from the time larvae began hatching till death

Survival of oncomiracidia varied between different aluminium concentrations and differences were significant (ANOVA *F*_(5, 38)_ = 7.44, *df* = 5, *P* < 0.0001). *Post-hoc* analysis of means for different concentrations indicated that differences were significant for comparisons between control with 60 μg Al/l (*F* = 2.04, *P* = 0.002) and 120 μg Al/l (*F* = 2.6, *P* < 0.0001) exposures; 7.5 μg Al/l with 60 μg Al/l (*F* = 1.32, *P* = 0.0023) and 120 μg Al/l (*F* = 1.88, *P* = 0.003); 15 μg Al/l with 120 μg Al/l (*F* = 1.63, *P* = 0.013); 30 μg Al/l with 120 μg Al/l (*F* = 1.57, *P* = 0.023). No significant differences in survival of oncomiracidia were found between low concentrations (0–30 μg Al/l) and 60 μg Al/l with 120 μg Al/l (all *P* > 0.05). Kaplan-Meier analysis of survival data (Table 1) indicated that the mean survival time for oncomiracidia decreased as aluminium concentration increased. Longest mean survival time of oncomiracidia was three days for control solutions while shortest survival time, which was less than one day, for 120 μg Al/l exposure (Fig. 5). Survival of oncomiracidia exposed to 7.5 μg Al/l–30 μg Al/l was similar and did not differ significantly. The log-rank test for survival indicated that there is a significant difference in the survival times of oncomiracidia for different exposures (Chi-square test *χ*^2^ = 36.1, *df* = 5, *P* < 0.0001). Therefore, oncomiracidia exposed to high levels of aluminium are more likely to die sooner than oncomiracidia exposed to 0–15 μg Al/l, indicating reduced longevity of the free swimming larvae.

**Table 1:** Kaplan-Meier analysis of the mean survival of oncomiracidia of *Paradiplozoon ichthyoxanthon* exposed to differing concentrations of aluminium

Concentration (μg Al/l)	Estimated mean survival (days) Mean time ± SEM	95 % confidence interval	
		Upper bound	Lower bound
Control	3.00 ± 0.82	1.40	4.60
7.5	1.88 ± 0.40	1.10	2.66
15	1.63 ± 0.26	1.11	2.41
30	1.57 ± 0.30	0.99	2.15
60	0.56 ± 0.24	0.08	1.03
120	0.00 ± 0.00	0	0

Abbreviation: *SEM* standard error of the mean

## Discussion

Monogeneans are particularly sensitive toward heavy metals in the environment [7]. The responses of diplozoids toward pollution, like other monogeneans, varies with the type of pollutant present [10, 14, 27, 28]. The present study highlighted the effect of varying nominal concentrations of aluminium on the hatchability of eggs and survival of oncomiracidia of *Paradiplozoon ichthyoxanthon* which has not been investigated previously. Comparatively, the hatchability of eggs was affected to a lesser degree than the survival of oncomiracidia when exposed to elevated (60–120 μg Al/l) concentrations of aluminium. Concerning the hatchability of helminth eggs, few studies have documented the effect of metals on the hatchability of these parasite eggs. Even though most eggs hatched in the present experiment, the variation in the hatchability of eggs exposed to aluminium revealed a time and concentration dependent effect. In terms of time, it would appear that the hatching time was accelerated in 30 μg Al/l and 60 μg Al/l media compared to control exposures and delayed in 120 μg Al/l exposure. Overall, there was little effect of aluminium on the percentage of eggs hatching in 7.5 μg/l–60 μg/l exposures compared to controls, while the opposite was observed for 120 μg/l which had the lowest percentage of hatched eggs. Hatchability, therefore, decreased in a concentration dependent manner. Morley et al. [29] indicated that exposure to elevated Cd and Zn concentrations (100–10,000 μg Cd/l and Zn/l) lowered the hatchability of eggs of the trematode, *Schistosoma mansoni*. Whereas, Khalil et al. [30] found that exposure to high Cd concentrations (10,000 μg/l) had little effect on the hatching of the eggs of the cestode, *Bothriocephalus acheilognathi*, but instead affected the survival of hatched coracidia.

The high percentage of eggs hatching in 7.5–60 μg Al/l exposures suggests that the eggs of *P. ichthyoxanthon* are impermeable to aluminium at low concentrations. However, this appeared to change in eggs containing almost fully developed embryos exposed to 120 μg Al/l where the highest percentage of perished, unhatched larvae were present. This is further supported by the observation that embryos in 120 μg Al/l exposure had reached the final stages of development where eye-spots and cilia were clearly visible [26]. This could be linked to alterations in the permeability of the egg capsule over the developmental period of the oncomiracidia instead of the resistance of embryos decreasing, where it would then be expected that even at low concentrations the unhatched oncomiracidia would have perished before or shortly after hatching. Such reduced survival of larvae at the final stage of development could therefore be due to thinning of egg shell allowing diffusion of the metal into the egg. In diplozoids localised thinning of the egg shell takes place at the anterior aspect of the egg shell, which forms the operculum [31]. This localised thinning at the operculum could allow and limit the entry of the metal into the egg, resulting in decreased survival of larvae exposed to high aluminium concentrations towards the end of development. Such thinning may account for the finding that larvae inside the eggs were fully developed and only died when exposed to 120 μg Al/l but in all other exposure concentrations death ensued only after the larvae had completely hatched. This finding corroborates with Thoney [32], who found that eggs of the monogenean, *Benedeniella posterocolpa*, became more susceptible toward exposure to trichlorfon and praziquantel in later developmental stages, and related this to increased permeability of the egg due to thinning of the egg shell.

The time taken for hatching of eggs of *P. ichthyoxanthon* to commence was delayed in 120 μg Al/l exposure, while exposure to 30 μg Al/l and 60 μg Al/l appeared to accelerate hatching time of eggs. According to Avenant-Oldewage & Milne [26] eggs of *P. ichthyoxanthon* incubated at 18 °C took 21 days for hatching to commence. MacDonald & Jones [24] found that hatching of eggs of *Diplozoon paradoxum* occurred 5–6 days after laying at temperatures ranging from 8–27 °C, but indicated that hatching was related to photoperiod and not temperature. Eggs in the current study were incubated in aluminium at a mean temperature (± SD) of 20.8 °C (± 0.21) which is similar to MacDonald & Jones [24], but compared to this study, eggs of *P. ichthyoxanthon* took longer to hatch which corroborates with the hypothesis that temperature has little effect on the hatching of monogenean eggs. This could have resulted in acceleration of the developmental time in *P. ichthyoxanthon* compared to Avenant-Oldewage & Milne [26] who reported hatching after 21 days at 18 °C with an 11 h lighting cycle. However, further assessment into the effect of temperature is required to validate this as in the current study temperature remained constant.

This became very apparent after larvae had hatched and were released from the egg shell in 60 μg/l–120 μg/l exposures, where survival time was markedly reduced. The egg shell, therefore, provides a degree of protection to the developing embryo by limiting the entry of metal into the egg, but as developmental completion is reached permeability increases. This is evident from the fact that although the lowest percentage of eggs to have hatched were present in 120 μg Al/l exposure, some eggs were still able to hatch and those that did not hatch contained fully developed embryos. Furthermore, 100 % of eggs exposed to 60 μg Al/l hatched, but death of larvae ensued rapidly.

Death of oncomiracidia in control media and lower exposure concentrations can be related to the depletion of yolk reserves which supply enough energy for the larvae to survive until infecting a host. Compared to the survival of oncomiracidia in control media it would appear that the larvae are sensitive toward all aluminium concentrations as evident by the concentration dependent decrease in survival. This has further implications on the dispersal of parasites within natural ecosystems and infection of new hosts as many parasites rely on larval stages for this vital function which could become disrupted by exposure to unfavourable environmental conditions. Zharikova [11] suggested that alterations in the abundance of *Diplozoon paradoxum* infecting the common bream (*Abramis brama*) was due to the effects of Cu toxicity on the free swimming oncomiracidia. Swimming activity of oncomiracidia of *P. ichthyoxanthon* was moderately affected by exposure to 7.5 μg Al/l, 15 μg Al/l and 60 μg Al/l, with the highest percentage of moribund larvae present in 60 μg/l exposure. Larvae in this state were found to not actively swim and even when agitated with a glass Pasteur pipette did not swim away or only swam a short distance before settling on the bottom of the beaker.

Reduced longevity and subsequent alterations in infrapopulation as a result of exposure to aluminium and zinc has been reported for *Gyrodactylus*. Poléo et al. [18] and Soleng et al. [19] both indicated a decline in the infrapopulation of adult *Gyrodactylus salaris* on salmon (*Salmo salar*) following exposure to 100–200 μg Al/l and 45 μg Al/l, respectively. Pettersen et al. [20] found that responses of two species of *Gyrodactylus* toward aluminium were different, but exposure to 200–206 μg Al/l resulted in complete elimination of infections by both. They determined that it took 72 h for complete elimination of *G. derjavini* infections and 187 h for elimination of *G. macronychus*. Gheorghiu et al. [33] found that exposure to 240 μg Zn/l was toxic toward *G. turnbulli* but exposure to 0–30 μg Zn/l was beneficial toward the parasite and infections increased compared to the control. Gheorghiu et al. [34] later found that decreases in infections by the same parasite exposed to varying concentrations of zinc was due to the toxic effect of the metal on parasite reproduction and survival of *in utero* F2 daughter parasites and impacted generation times of larvae in a concentration dependent manner.

## Conclusions

The current results indicate that oncomiracidia of *P. ichthyoxanthon* are sensitive toward elevated aluminium concentrations. Exposure to aluminium does not affect the development of embryos and the egg shell therefore functions as an effective barrier limiting the entry of aluminium into the egg. Hatchability was moderately affected by exposure to 7.5 μg Al/l–60 μg Al/l, but was reduced in the 120 μg Al/l exposure by resulting in death of fully developed embryos still within the eggs. This finding suggests that the permeability of eggs to aluminium penetration and possibly other metals changes as embryonic development progresses. The sensitivity of oncomiracidia toward metals increased in a concentration dependant manner and was evident from the reduced longevity in oncomiracidia exposed to 60 μg Al/l and 120 μg Al/l. Compared to an earlier field investigation by Gilbert & Avenant-Oldewage [10] in the Vaal River system where the local extinction of *P. ichthyoxanthon* at a site along the Vaal River was related to high metal concentrations, the current data suggests that compared to aluminium concentrations recorded for this site the survival of oncomiracidia and hatchability of the eggs would not have been significantly affected by aluminium alone. Rather, the fact that many other trace elements were found to be elevated in this study further suggests that the effects noted may be due to metals acting in synergy on the parasites. This further points to the complexity of biological and chemical interactions under natural conditions and, therefore, further exposure experiments incorporating other trace elements are required in order to better understand this relationship. These results further serve as support for the use of these parasites as sentinels for monitoring environmental pollution, but, further indicates that compared to other ecotoxicological investigations there is still a large gap in the knowledge with regard to the effects of metals on different life stages of parasites and related sensitivities of these organisms toward elevated concentrations.

## Abbreviations

Al, aluminium; RO, reverse osmosis
